# Marginal-Zone B-Cells Are Main Producers of IgM in Humans, and Are Reduced in Patients With Autoimmune Vasculitis

**DOI:** 10.3389/fimmu.2018.02242

**Published:** 2018-10-02

**Authors:** Daniel Appelgren, Per Eriksson, Jan Ernerudh, Mårten Segelmark

**Affiliations:** ^1^Department of Medical and Health Sciences, Linköping University, Linköping, Sweden; ^2^Department of Rheumatology, Linköping University, Linköping, Sweden; ^3^Department of Clinical Immunology and Transfusion Medicine, and Department of Clinical and Experimental Medicine, Linköping University, Linköping, Sweden; ^4^Department of Nephrology, Linköping University, Linköping, Sweden; ^5^Department of Clinical Sciences in Lund, Nephrology, Lund University and Skåne University Hospital, Lund, Sweden

**Keywords:** marginal-zone (MZ) B-cells, B1 B-cells, innate-like B-cells, natural antibodies, T-cell-independent antibodies, IgM, autoimmunity, anti-neutrophil cytoplasmic autantibody (ANCA) associated vasculitis

## Abstract

In mice, B1 and marginal zone (MZ) B-cells play an important role in prevention of autoimmunity through production of regulatory cytokines and natural antibodies. There is limited knowledge about the human counterparts of these cells. We therefore investigated functions of MZ-like B-cells and the frequency of circulating MZ-like and B1-like B-cells in healthy controls (HC), as well as in patients with autoimmune vasculitis to learn more about the role of these cells in autoimmune disease. After stimulation with CpG oligodeoxynucleotides (ODN) of class B *in vitro*, MZ-like B-cells were the main producers of IgM whereas switched memory B-cells primarily produced IgG and IgA. TNF and IL-10 were produced by both MZ-like and switched memory B-cells. Neither antibody nor TNF/IL-10 production by the B-cell subsets differed between patients and HC. Patients with autoimmune vasculitis, irrespective of disease activity, had lower percentage and absolute numbers of circulating MZ-like B-cells, and lower absolute numbers of B1-like B-cells. The percentage of B1-like B-cells was reduced during active disease. These findings remained significant when the analysis was confined to active treatment-naïve patients (disease onset).Our results suggest that human innate-like B-cells might have a physiological role in prevention of autoimmunity.

## Introduction

B-cells in mice are well characterized and generally divided into three subsets; B1, follicular and marginal zone (MZ) B-cells, where the two latter are also referred to as B2 B-cells (1). B1 B-cells produce antibodies spontaneously (natural antibodies) and toward T-cell-independent (TI) antigens, while follicular B-cells develop into plasma cells after maturation in B-cell follicles where they interact with T-cells after encountering their cognate antigen (i.e., T-cell-dependent (TD) antigens) (1, 2). MZ B-cells can, similar to B1 B-cells, produce natural antibodies and antibodies in response to TI antigens, but also to TD antigens (3, 4). B1 B-cells are, however, the major source of natural antibodies. Natural and TI antibodies show broad specificity and low affinity, and are often of IgM class (3, 5). This is in contrast to antibodies produced toward TD antigens, which are highly antigen specific, of high affinity, and of IgG, IgA, or IgE class (2, 6). B1 and MZ B-cells, both considered innate-like cells, have a limited antibody repertoire, recognizing mainly common bacterial motifs and self-antigens (1, 3). They are important in the early antibody-response toward invading pathogens, as well as in maintaining homeostasis through clearance of dead/dying cells and cellular debris (7, 8). Innate-like B-cells also exert regulatory features by production of IL-10 (9). Counterparts to mice B-cell subsets can be found also in humans. Maturation stages for follicular B-cells from naïve B-cells to plasma cells, as well as phenotypic changes during this process, are well-described, whereas there is yet no consensus on the developmental path or the characteristic phenotype of innate-like B-cells (10, 11). Nevertheless, CD27^+^IgD^+^IgM^high^ have been suggested as relevant markers for MZ-like B-cells and CD20^+^CD27^+^CD43^+^CD70^−^ for B1-like B-cells, as these cells harbor substantial functional similarities to the corresponding cells in mice (11). Whether the definition for B1-like B-cells really represents a truly similar population to B1 B-cells in mouse has, however, been debated (12, 13).

Central and peripheral tolerance mechanisms serve to prevent development of autoreactive lymphocytes and to limit strong reactions to self in the periphery (14). Autoimmune disease can emerge when lymphocytes escape these tolerance mechanisms, as this allows interactions of autoreactive B and T-cells, with production of pathogenic autoantibodies. B-cells producing pathogenic autoantibodies can stem from both autoreactive and non-autoreactive precursors (14). Prolonged exposure to self-antigens is connected to autoimmune disease development. Therefore, production of natural and T1 autoantibodies by innate-like B-cells and aiding in clearance of self-antigens are considered an important mechanism to prevent development of autoimmune disease (15–17).

Anti-neutrophil cytoplasmic autoantibody (ANCA)-associated vasculitis (AAV) constitute a group of autoimmune diseases characterized by pauci-immune necrotizing small-vessel inflammation and by the production autoantibodies against proteinase 3 (PR3-ANCA) and myeloperoxidase (MPO-ANCA) (18). AAV comprise granulomatous with polyangiitis (GPA), microscopic polyangiitis (MPA), and eosinophilic granulomatous with polyangiitis (EGPA) (18, 19). ANCA are implicated in the pathogenesis through activation of neutrophils by binding to PR3 or MPO on the cell surface (20–23). It has been reported that also healthy individuals harbor autoreactive PR3-specific B-cells (24), as well as circulating antibodies with similar specificity as PR3-ANCA and MPO-ANCA (25). This suggests that antibodies with ANCA reactivity belong to the repertoire of natural and TI antibodies. It has also been proposed that the pathogenicity of ANCA vary with disease activity (23, 26), further indicating that not all ANCA are pathogenic. AAV is a relapsing and remitting disease, and there are several indications that remission in AAV is an active state where a subclinical disease process is kept under control by regulatory mechanisms. Thus, defective regulatory mechanisms may increase disease activity, as well as may dispose for the development of disease in the first place.

To extend the knowledge of humans innate-like B-cells, as well as their role in autoimmune disease, we investigated the production of various antibody subclasses (IgM, IgG, and IgA) and cytokines (TNF and IL-10) by MZ-like B-cells in healthy controls (HC) and in in patients with autoimmune vasculitis. We also used multi-color flow cytometry to investigate the frequency of circulating MZ-like and B1-like B-cells, and we related our findings to disease activity, treatment and autoantibody levels.

## Material and methods

### Study population

Patient and HC study populations were recruited at the Departments of Rheumatology and Nephrology at the University Hospital in Linköping, Sweden. For the functional study of B-cells, 7 AAV patients (all GPA) and 8 HC were included. All patients were in remission (Birmingham vasculitis activity score (BVAS) of 0) (Table 1). For the B-cell phenotype study, 64 AAV patients and 31 HC were included. 34 patients were in remission and 28 patients had active disease (Table 2). Patients previously treated with rituximab (RTX) and less than 0.5% B-cells within the lymphocyte population were excluded from this study. Patients in both cohorts were diagnosed with AAV according to the European Medicines Agency algorithm (27). Data on disease activity, disease extension, treatment and ANCA serology for both cohorts was obtained from medical records. This study was approved by the Regional Ethical Review Board in Linköping. All subjects gave written informed consent in accordance with the Declaration of Helsinki.

**Table 1:** Study population for B-cell functional studies.

	**Healthy controls**	**AAV remission**	***p*-value**
Number of individuals: *n* (female/male)	8 (3/5)	7 (2/5)	1.00
Age: *median years* (range)	69 (68–71)	69 (38–84)	0.845
ANCA at diagnosis: *PR3/MPO*	na	7/0	–
*GPA/MPA*	na	7/0	–
Lymphocytes: *median x10^9^/L* (IQR)	1.8 (1.6–2.7)	1.4 (1.2–1.9)	0.186
Creatinine: *median μmol/L* (range)	83 (52–113)	91 (82–123)	0.244
C-reactive protein: *median mg/L* (range)	<5	<5 (<5–7)	–
**TREATMENT AT TIME OF SAMPLING:**			
Prednisolone, *n* (%)	na	5 (71)	–
Prednisolone, *median mg/day* (range)	na	2.5 (0–3.8)	–
Azathioprine, *n* (%)	na	1 (14)	–
Methotrexate, *n* (%)	na	3 (43)	–
No immunosuppressive therapy, *n (%)*	na	0 (0)	–
**PRIOR TREATMENT:**			
Rituximab: *n* (%)	na	2 (29)	–
Time since rituximab at sampling in treated patients: *median months* (range)	na	66 (43–90)	–

*AAV, ANCA-associated vasculitis; PR3, proteinase 3; MPO, myeloperoxidase; GPA, granulomatous with polyangiitis; MPA, microscopic polyangiitis; na, not applicable*.

*Fisher's exact test was used to compare discrete variables and Mann-Whitney U test for continuous variables bwteen the groups*.

*Significance assumed at p < 0.05*.

**Table 2:** Study population for B-cell phenotyping.

	**Healthy control**	**AAV remission**	**AAV active**	***p*-value**
Number of individuals: *n* (female/male)	31 (17/14)	34 (19/15)	28 (11/17)	0.361
Age: *median years* (range)	60 (26–85)	69 (22–87)	65 (29–93)	0.085
ANCA at diagnosis: *PR3/MPO*	na	25/9	17/11	0.413
*GPA/MPA*	na	25/9	19/9	0.779
Disease *onset/relapse*	na	na	19/9	–
**ORGAN INVOLVEMENT:**				
ENT, *n* (%) [*ever/sample*]	na	23 (68) [*23/na*]	14 (50) [1*4/14*]	0.198
Lungs, *n* (%) [*ever/sample*]	na	14 (41) [*14/na*]	14 (50) [*14/9*]	0.610
Kidneyes, *n* (%) [*ever/sample*]	na	19 (56) [*19/na*]	21 (75) [*21/16*]	0.182
Nervous system, *n* (%) [*ever/sample*]	na	10 (29) [*10/na*]	10 (36) [*10/6*]	0.785
**TREATMENT AT TIME OF SAMPLING:**				
Prednisolone, *n* (%)	na	22 (65)	14 (50)	0.345
Prednisolone, median *mg/day* (range)	na	2.5 (0–13)	1.3 (0–30)	0.979
Azathioprine, *n* (%)	na	8 (24)	1 (3.6)	0.033
Methotrexate, *n* (%)	na	10 (29)	3 (11)	0.116
Mycophenolate mofetil *n* (%)	na	4 (12)	1 (3.6)	0.366
No immunosuppressive therapy, *n (%)*	na	3 (9)	14 (50)	0.0004
**PRIOR TREATMENT:**				
Cyclophosphamide: *n* (%)	na	30 (88)	7 (25)	< 0.0001
Rituximab: *n* (%)	na	11 (32)	5 (18)	0.250
Time since rituximab at sampling in treated patients: median *months* (range)	na	26 (7–86)	18 (11–94)	0.935
Neither cyclophosphamide or rituximab: *n* (%)	na	2 (6)	20 (71)	< 0.0001
BVAS: *median* (range)	na	0	14 (2–26)	–

*AAV, ANCA-associated vasculitis; PR3, proteinase 3; MPO, myeloperoxidase; GPA, granulomatous with polyangiitis; MPA, microscopic polyangiitis; ENT, ear, nose and throat; BVAS, Birmingham vasculitis activity score; na, not applicable*.

*Fisher's exact test or the Chi-squared test were used to compare discrete variables and Mann-Whitney U test or the Kruskal-wallis test followed by Dunn's multiple comparison test were used to compare continuous variables between the groups*.

*Significance assumed at p < 0.05*.

### Isolation of peripheral blood mononuclear cells (PBMC) and B-cells

Blood was drawn into heparin-treated tubes (Terumo Europe N.V., Leuven, Belgium). White blood cell count (WBC) and absolute number of circulating lymphocytes were analyzed at the Clinical Chemistry Laboratory at the Linköping University Hospital (Cell-Dyn Sapphire; Abbot, Abbot Park, IL, USA). PBMC were isolated through gradient centrifugation on Ficoll (GE Healthcare, Uppsala, Sweden). PBMCs for phenotype analyses were resuspended in freezing medium containing 90% fetal bovine serum (FBS)(GE Healthcare) and 10% dimethyl sulfoxide (Sigma-Aldrich, St Louis, MO, USA), placed in a room tempered Mr. Frosty Freezing Container (Thermo Fisher Scientific, Waltham, MA USA) and subsequently put in a −70°C freezer for 24–48 h. Samples were then transferred for cryopreservation in liquid nitrogen. B-cells to be used for functional studies were enriched from fresh PBMCs by magnetic-activated cell sorting, through positive selection using CD19 MicroBeads (Miltenyi Biotec, Bergisch Gladbach, Germany), before isolation of B-cell subsets (see section Fluorescence Activated Cell Sorting (FACS) of B-cell Subsets for Functional Studies).

### Flow cytometry for B-cell phenotyping

For phenotype analyses, PBMCs were thawed in a 37°C water bath before being washed twice in 9 ml RPMI 1640 (Gibco Paisley, UK) + 10% FBS (first wash in 37°C medium and the second wash in room tempered medium). Viability of the cells after thawing was controlled with trypan blue dye exclusion and exceeded 90 % in the majority of the samples. Cells were resuspended in PBS + 0.1% FBS before labeling of surface molecules to compare the proportions and levels of switched memory (SwMe) B-cells (CD19^+^CD27^+^IgD^−^), non-switched memory (NSM) B-cells (CD19^+^CD27^+^, IgD^+^), naïve B-cells (CD19^+^CD27^−^IgD^+^), double negative (DN) B-cells (CD19^+^CD27^−^IgD^−^), MZ-like B-cells (CD19^+^CD27^+^IgD^+^IgM^high^), and B1-like B-cells (CD20^+^CD27^+^CD43^+^CD70^−^) (Figure S1). FMO-controls or FMO-controls combined with isotype-controls were used to set appropriate gates to determine positivity for a specific surface molecule. IgD-V500 was bought from BD Biosciences (San Jose, CA, USA) whereas the rest of the antibodies were bought from BioLegend (San Diego, CA, USA). The samples were run on a Gallios Flow Cytometer (Beckman Coulter, Brea, CA, USA) at medium speed to avoid turbulence, and ~1,000 events /second were registered. The freezing and thawing procedure did not affect the proportions of the B-cell subsets investigated (HC, *n* = 4) (data not shown). Absolute numbers of B-cell subsets were based on the proportion (%) of B-cells within the lymphocyte population combined with the absolute number of lymphocytes from the WBC.

### Fluorescence activated cell sorting (FACS) of B-cell subsets for functional studies

For the functional studies we included CD45RB in our gating strategy to in more detail distinguish SwMe B-cells, naïve B-cells and MZ-like B-cells (11). Fresh enriched B-cells were resuspended in PBS + 0.1% FBS and labeled with antibodies to determine SwMe B-cells (CD19^+^CD27^+^IgD^−^CD45RB^high^), naïve B-cells (CD19^+^CD27^−^IgD^−^CD45RB^low^) and MZ-like B-cells (CD19^+^CD27^+^IgD^+^IgM^high^CD45RB^high^). Cells were also labeled for CD3 to avoid T-cell contamination during sorting. FMO-controls or FMO-controls combined with isotype-controls were used to set appropriate gates to determine positivity for a specific surface molecule. IgD-VH500 was bought from BD Biosciences and CD45RB from Thermo Fisher (Rockford, IL, USA), whereas the other antibodies were bought from BioLegend. B-cells were resuspended at 2.5 × 10^6^ cells /ml in PBS + 2% FBS before sorting on a BD FACSARIA III (BD Biosciences). Sorting was performed using a 100 μm nozzle at a rate of ~2,000 events /s. Sorted B-cells were collected in FBS-coated 5 ml flow cytometry tubes containing 1 ml RPMI 1640 + 10% FBS. B-cell subsets were reanalysed in annexin V binding buffer (BD Biosciences; diluted 1:10 in distilled water) together with annexin V (Biolegend) to evaluate cell viability. Cell viability was generally good for both HC and AAV patients [HC median MZ-like B-cells 89% (range 86–92), SwMe B-cells 90% (range 88–95), and Naïve B-cells 90% (range 86–95), and AAV median MZ-like B-cells 88% (range 86–98), SwMe B-cells 92% (range 92–98), and Naïve B-cells 88% (range 86–92)]. Purity of the different subsets was consistently high [HC median MZ-like B-cells 94% (range 91–97), SwMe B-cells 98% (range 97–100), and Naïve B-cells 99% (range 98–100), and AAV median MZ-like B-cells 95% (range 91–99), SwMe B-cells 98% (range 97–100), and Naïve B-cells 97% (range 93–100)], except during isolation of Naïve B-cells from two patients where there were contaminations of SwMe B-cells, resulting in Naïve B-cell purity of 54 and 83%. These two naïve B-cell samples were therefore excluded from the study.

### Measurement of antibody production with ELISA

Sorted B-cell subsets were resuspended to 50 × 10^3^ cells /ml in RPMI 1,640 supplemented with 10% FBS and 1% penicillin/streptomycin, and cultured for 5 days at 37°C and 5% CO_2_, either in the presence of 1 μg/ml CpG oligodeoxynucleotides (ODN) of class B (CpG-B ODN, ODN 2006; Invivogen, San Diego, CA, USA) or without stimulation. Cells were then centrifugated and supernatants collected. Ninety-six-well medisorp plates (Thermo Fisher) were coated over night at 4°C with 10 μg/ml anti-IgM (Dako, Santa Clara, CA, USA), 10 μg/ml anti-IgA (Dako), and with 2.5 μg/ml anti-IgG antibodies (Mabtech, Stockholm, Sweden). For the IgG ELISA, a blocking step was carried out the next day for 1 h with PBS + 0.05% Tween 20 + 0.1% FBS. 13-point standard curves ranging from 250 to 0.313 ng/ml were used for all ELISAs. Standards and samples (diluted 1:4 in all ELISA) in duplicates were incubated for 2 h in room temperature. After washing, HRP-conjugated anti-IgM (1:1,000) (Dako) and anti-IgA (1:4,000) (Dako) antibodies, for the IgM and IgA ELISA respectively, were added for 2 h in room temperature. After another washing step, tetramethylbenzidine (TMB) was added for 8 min followed by adding the H_2_SO_4_ stop solution. Regarding the IgG ELISA, after incubation with standards and samples and a subsequent washing step, these plates were incubated with alkaline phosphatase (ALP)-conjugated anti-IgG antibodies (Mabtech) for 2 h in room temperature. After another washing step, a phosphatase substrate for ALP (Sigma Aldrich) was added and the plates were incubated 40 min before reading. IgG ELISA plates were read at 405 nm and IgM and IgA ELISA plates at 450 nm in a VersaMax ELISA microplate reader (Molecular Devices, Sunnyvale, CA, USA).

### ELISPOT to determine production of IL-10 and TNF

The Human TNF-α ELISpot ^BASIC^ (ALP) and Human IL-10 ELISpot ^BASIC^ (ALP) kits (Mabtech) were used to measure the amount of TNF and IL-10 producing cells, respectively, according to the manufactures instructions. Briefly, 96-well plates with polyvinylidene difluoride-membrane (Merck Millipore, Burlington, MA, USA) were treated with 35% ethanol for 1 min and then coated with 15 μg/ml anti-IL-10 or anti-TNF antibodies (Mabtech) over night at 4°C. The plates were then blocked with RPMI 1640 + 10% FBS for 1 h in room temperature. Sorted B-cell subsets were resuspended to 50 × 10^3^ cells /ml in RPMI 1640 supplemented with 10% FBS and 1% penicillin/streptomycin, and cultured for 36 h at 37°C and 5% CO_2_, either in the presence of 10 μg/ml CpG-B ODN (Invivogen) or without stimulation. For detection of spots after 36 h, plates were incubated with detection antibodies for TNF (0.5 μg/ml) or IL-10 (1μg/ml) (Mabtech) for 2 h in room temperature. Streptavidin-ALP antibodies (1:1000) (Mabtech) were then allowed to bind for 1 h in room temperature, before TMB was added for spot development. When distinct spots had occurred, after 10 min for all plates, color development was stopped by rinsing the plate extensively with deionised water. The plates were left to dry before being analyzed in an automated process using a Bioreader 5000 Pro-F for ELISPOT plates (Bio-Sys GmbH, Karben, Germany). A spot-size of 10 μm was used as cut-off.

### Statistics

Data were analyzed using GraphPad Prism, version 6.02 (GraphPad Software, San Diego, CA, USA). Fisher's exact test and the chi-square test were used to compare discrete variables, in 2 × 2 and 3 × 2 contingency tables, respectively. D'Agostino and Pearson omnibus normality tests were used to determine whether the data on continues variables had a Gaussian distribution or not. All data had a non-Gaussian distribution and therefore the Kruskal-Wallis test followed by Dunn's multiple comparison test was used when comparing more than two groups with independent observations, and Mann-Whitney U test was applied in comparisons between two groups with independent observations. Data are presented as median and interquartile range (IQR). For correlation analyses, Spearman's rank correlation coefficient (r_s_) was calculated. A *P*-value < 0.05 was considered statistically significant in all analyses.

## Results

### MZ-like B-cells produce antibodies of IgM class, as well as TNF and IL-10

**Figure 1:**
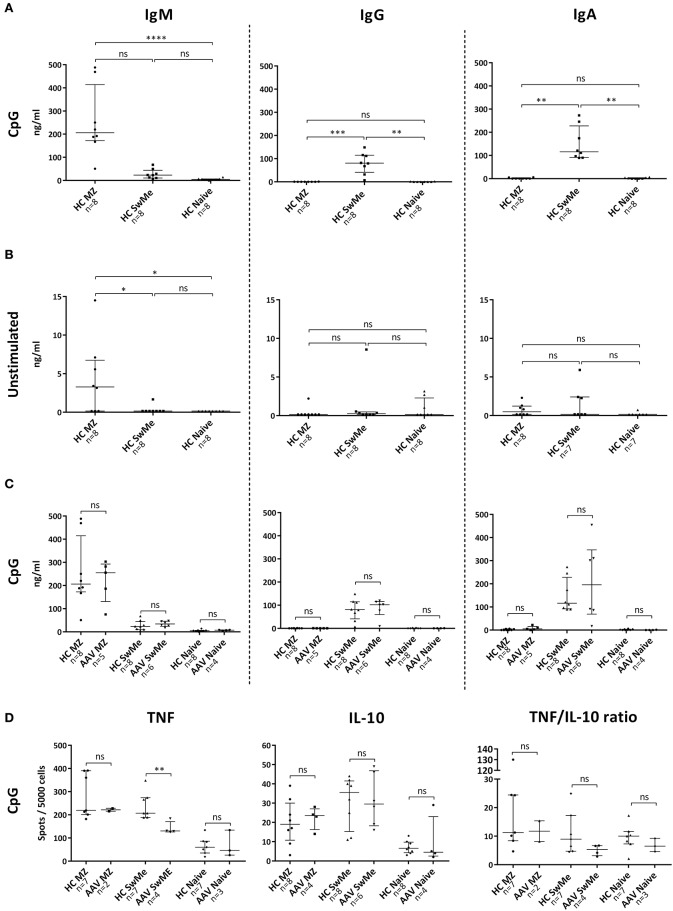
Antibody and cytokine production *in vitro* by various B-cell subsets. **(A)** Stimulation of B-cells from healthy controls (HC) with CpG-B oligodeoxynucleotides (ODN) showed that marginal zone (MZ)-like B-cells are the main producers of IgM, whereas switched memory (SwMe) B-cells primarily produces IgG and IgA. **(B)** Spontaneous Ig production was generally scarce among the different B-cell subsets, but MZ-like B-cells produced IgM spontaneously. **(C)** B-cells from patients in remission behaved very similar to those from HC concerning antibody production after CpG-B ODN stimulation, with no significant differences between the groups. **(D)** MZ-like and SwMe B-cells produced most TNF and IL-10 after CpG-B ODN stimulation, but SwMe B-cells from patients produced TNF to a lower extent than SwMe B-cells from HC. There were, however, no differences in the TNF/IL-10 ratio among the various B-cell subsets in patients and HC. Kruskal-Wallis test followed by Dunn's multiple comparison test was used to compare more than two groups with independent observations **(A,B)**, and Mann-Whitney U test was applied in comparisons between two groups with independent observations **(C,D)**. Bars indicate median and inter-quartile range. *p < 0.05, **p < 0.01, ***p < 0.001, ****p < 0.0001.

In order to evaluate functional properties of human MZ-like B-cells, cytokine and antibody production was measured *in vitro* and compared with that of naïve and SwMe B-cells. Upon CpG-B ODN stimulation of cells from HC, MZ-like B-cells produced IgM, while SwMe B-cells primarily produced IgG and IgA. Naïve B-cells, on the other hand, did not produce any of the studied antibody subclasses despite the stimulation (Figure 1A). MZ-like B-cells showed some spontaneous production of IgM, while spontaneous production was low for all Ig subclasses by the other B-cell subsets (Figure 1B). Ig production was also measured from the corresponding cells in patients with autoimmune vasculitis, all of whom had low-dose maintenance treatment and were PR3-ANCA positive at diagnosis (Table 1). B-cell subsets from patients produced similar amounts of antibodies as those from HC upon CpG-B ODN stimulation (Figure 1C), as well as spontaneously (data not shown). Both MZ-like and SwMe B-cells, and to some extent naïve B-cells, produced TNF and IL-10 after CpG-B ODN stimulation (Figure 1D). Some MZ-like and SwMe B-cells also produced IL-10 spontaneously, whereas TNF was produced spontaneously by all B-cell subsets (Figure S3). SwMe B-cells from patients produced TNF to a lower extent than those in HC (Figure 1D), but the ratio of TNF and IL-10 production for the various B-cell subsets did, however, not differ significantly between patients and HC (Figure 1D).

### Proportions and absolute numbers of total B-cells

A total of 62 patients were included in the phenotype study. No patient was sampled twice. Patients either had GPA or MPA. Patients with active disease (*n* = 28) were all sampled before starting induction therapy with cyclophosphamide or rituximab (RTX), and most of them were on no or only low steroid doses (Table 2). Among the patients with active disease, 18 had newly diagnosed disease, while 10 were sampled at the time of a relapse. Most of the 34 patients sampled in remission were on maintenance therapy and 11 of them previously received RTX, with a median time from dosing to sampling of 26 months (Table 2). Lymphocyte counts were lower during both active disease and remission compared with HC [median active 1.2 × 10^9^/l (IQR 0.8–1.1.9 × 10^9^) and remission 1.6 × 10^9^/l (IQR 1.2–2) vs. HC 2.0 (IQR 1.7–2.4 × 10^9^/l), *p* < 0.0001 and *p* = 0.0184, respectively]. However, the percentage of B-cells within the lymphocyte population did not differ between the groups [median active 5.3% (IQR 2.6–7.5) vs. remission 5.3% (IQR 2.3–6.6)] vs. HC 5.1% (IQR 3.8–7.5).

### Reduced frequencies of innate-like B-cells in AAV patients

**Figure 2:**
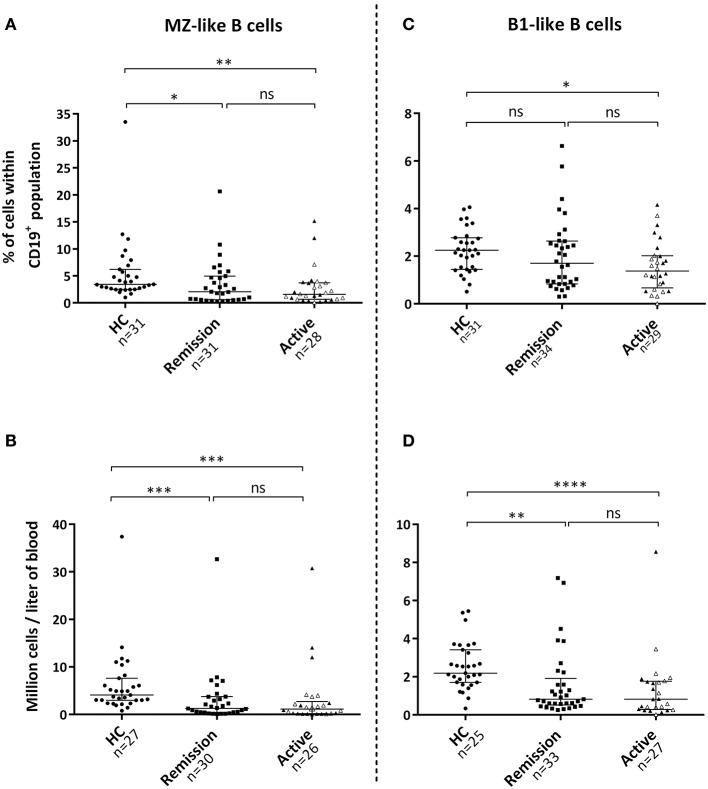
Levels of marginal zone (MZ)-like and B1-like B-cells in the blood circulation. **(A)** Patients with active disease and patients in remission exhibited a reduced percentage of MZ-like B-cells within the B-cell population, **(B)** as well as lower absolute numbers of these cells in the circulation compared with healthy controls. **(C,D)** Percentage of B1-like B-cells was only reduced during active disease, whereas absolute numbers were lower in both patient groups. Gray triangles represent 14 treatment-naïve patients with active disease. Kruskal-Wallis test followed by Dunn's multiple comparison test was used to compare more than two groups with independent observations **(A–D)**. Bars indicate median and inter-quartile range. *p < 0.05, **p < 0.01, ***p < 0.001, ****p < 0.001.

The proportion of MZ-like B-cells within the B-cell population, as well as absolute numbers of these cells, were reduced in the circulation during active disease and remission compared with HC (Figures 2A,B). The proportion of B1-like B-cells within the B-cell population was also reduced in patients with active disease, whereas absolute numbers of these cells were lower in patients irrespective of disease activity (Figures 2C,D). Patients with active disease possessed a lower percentage of SwMe B-cells within the B-cell population compared with HCs (Figure S2). Absolute numbers of both SwMe B-cells and NSM B-cells were reduced in patients irrespective of disease activity (Figure S2).

### Influence of age and sex on B-cell subsets

There were no significant differences in B-cell subsets between women and men among HC (data not shown). Both proportion and absolute numbers of B1-like B-cells correlated negatively with age in HC (rs = −0.39, *p* = 0.029, and rs = −0.36, *p* = 0.045, respectively). Although not statistically significant, the proportion and numbers of MZ-like B-cells also tended to decrease with age in HC (rs = −0.34, *p* = 0.058, and rs = −0.33, *p* = 0.073, respectively]. There were, however, no significant differences in age between the groups, although HC were slightly younger [median active 65 years (IQR 59–78) vs. remission 69 years (IQR 48–77) vs. HC 60 years (IQR 56–65)].

### Effect of treatment on B-cell subsets

#### Active disease

To assure that our findings on B-cell subsets in patients with active disease were not caused by previously given treatment, we compared the results from the subgroup of patients who were treatment-naïve (disease onset) with data from HC (Table 3). These active patients had reduced proportions and absolute numbers of B1-like, MZ-like and SwMe B-cells, and reduced numbers of NSM B-cells, compared with HC, results which are very similar to the analyses including all active patients (Figure 1 and Figure S2). Additionally, they had reduced proportions of NSM B-cells and an increased proportion of DN B-cells (Table S1).

**Table 3:** Comparison of B-cell subsets in active treatment-naïve AAV patients with HC.

	**% of B-cells *****median***** (IQR)**			**Absolute numbers *****x 10**^**6**^**/l (IQR)***		
**Subsets**	**Active (*n* = 14)**	**HC (*n* = 30-31)**	***p*-value**	**Active (*n* = 12)**	**HC (*n* = 30-31)**	***p-*value**
B-cells (CD19^+^)	6.9^*^ (4.4–8.6)	5.0^*^ (3.8–7.5)	0.2258	103 (49–134)	104 (79–164)	0.4858
MZ-like B-cells	1.8 (0.86–3.3)	3.4 (2.5–6.2)	0.0014	1.6 (0.61–3.4)	4.1 (3.0–7.6)	0.0003
B1-like B-cells	1.8 (0.46–1.8)	2.3 (1.4–2.8)	0.0013	0.87 (0.41–1.9)	2.2 (1.7–3.4)	0.0013

* *Proportion of total B-cells (CD19^+^) within the lymphocyte population*.

*AAV, ANCA-associated vasculitis; HC, healthy controls; MZ, marginal zone; IQR, interquartile range*.

*Mann-Whitney U test was used to compare two groups with independent observations. P < 0.05 is statistically significant*.

#### Remission

##### Rituximab

As RTX treatment is known to affect both numbers and the composition of B-cells, we evaluated the impact of this treatment on our patients in remission. Although the percentage and absolute numbers of the whole B-cell population was not significantly reduced in RTX treated patients (Table 4), both proportions and absolute numbers of the MZ-like B cell subset, as well as absolute numbers of the B1-like B-cell subset were reduced in this treatment group (Table 4). After exclusion of RTX treated patients there were no longer any significant differences in the proportions of MZ-like B-cells between AAV patients in remission and HC (Table 4).

**Table 4:** Effect of RTX treatment in AAV patients in remission on B-cell subsets.

	% of B-cells^*^median (IQR)			**Absolute numbers x 10**^**6**^**/l (IQR)**		
**Subsets**	**No RTX (*****n*** = **19–23)**	**RTX (*****n*** = **10–11)**	***p*****-value**	**No RTX (*****n*** = **19-22)**	**RTX (*****n*** = **10-11)**	**p-value**
**PATIENTS IN REMISSION (NO RTX vs. RTX)**						
B-cells (CD19^+^)	5.7^*^ (3.1–6.8)	2.9^*^ (1.8–5.9)	0.1720	90 (35–146)	38 (24–106)	0.2425
MZ-like B-cells	3.3 (1.3–6.2)	0.59 (0.40–1.8)	0.0049	2.2 (0.89–5.7)	0.24 (0.15–1.4)	0.0013
B1-like B-cells	1.8 (0.93–2.9)	0.96 (0.83–2.5)	0.4399	1.0 (0.69–2.4)	0.57 (0.33–1.6)	0.0042
**Subsets**	**No RTX (*****n*** = **19–23)**	**HC (*****n*** = **30–31)**	***p-*****value**	**No RTX (*****n*** = **19–22)**	**HC (*****n*** = **30-31)**	***p*****-value**
**PATIENTS IN REMISSION VS. HC**						
B-cells (CD19^+^)	5.7^*^ (3.1–6.8)	5.0^*^ (3.8–7.5)	0.7981	90 (35–146)	104 (79–164)	0.0943
MZ-like B-cells	3.3 (1.3–6.2)	3.4 (2.5–6.2)	0.4219	2.2 (0.89–5.7)	4.1 (3.0–7.6)	0.0193
B1-like B-cells	1.8 (0.93–2.9)	2.3 (1.4–2.8)	0.3872	1.0 (0.69–2.4)	2.2 (1.7–3.4)	0.0056

* *Proportion of total B-cells (CD19^+^) within the lymphocyte population*.

*RTX, rituximab; AAV, ANCA-associated vasculitis; HC, healthy controls; MZ, marginal zone; IQR, interquartile range*.

*Mann-Whitney U test was used to compare two groups with independent observations. P < 0.05 is statistically significant*.

##### Corticosteroid therapy

Possible effects of corticosteroid therapy on the B-cell homeostasis were evaluated in the 23 patients in remission who had previously not been treated with RTX. 16 of the 23 patients had corticosteroid therapy at sampling (Table 1). The prednisolone dose (mg/day) correlated negatively with absolute numbers of B-cells and the following subpopulations: B1-like B-cells [r_s_ = −0.53, *p* = 0.0011], NSM B-cells [r_s_ = −0.63, *p* = 0.0029] and Naïve B-cells [r_s_ = −0.61, *p* = 0.0042].Similar results, but not statistically significant, were seen also for MZ-like B-cells [r_s_ = −0.44, *p* = 0.051] and SwMe B-cells [r_s_ = −0.4, *p* = 0.0539], but not for DN B-cells [r_s_ = −0.31, *p* = 0.17]. DN B-cells was the only population whre the proportions of cells changed with prednisolne treatment [r_s_ = 0.60, *p* = 0.0044]. Effect of other immune modulating treatments [azathioprine (*n* = 5), methotrexate (*n* = 4) or mycophenolate mofetil (*n* = 4)] was not possible to investigate due to too small sample sizes.

### Relation between innate-like B-cells and ANCA levels and birmingham vasculitis activity score (BVAS)

**Figure 3:**
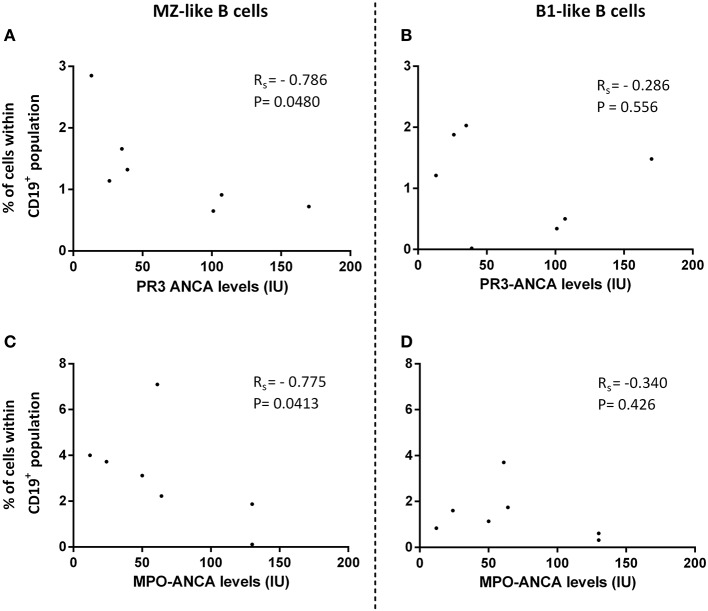
Innate-like B-cells and ANCA levels in active treatment-naïve patients. **(A,B)** Proportions of marginal-zone (MZ)-like B-cells, **(C,D)** but not B1-like B-cells, correlated negatively with PR3-ANCA and MPO-ANCA levels in active treatment-naïve patients. Spearman's rank correlation coefficient (r_s_) was calculated. *P* < 0.05 is statistically significant.

For patients with active disease, 17 were PR3-ANCA positive and 11 MPO-ANCA positive (Table 1). Within the group of active patients, treatment-naïve patients consisted of 7 patients that were PR3-ANCA positive at diagnosis, and 7 that were MPO-ANCA positive. When correlating ANCA levels with the proportions of innate-like B-cell populations in the group of active treatment-naïve patients, we observed negative correlations between both PR3-ANCA and MPO-ANCA with MZ-like B-cells, but not B1-like B-cells (Figure 3). Proportions of B1-like B-cells did, however, correlate negatively with birmingham vasculitis activity score (BVAS) in these patients (*n* = 14) [B1-like B-cells r_s_ = −0.55, *p* = 0.0037 and MZ-like B-cells r_s_ = −0.45, *p* = 0.100].

## Discussion

Natural antibodies and other antibodies produced without T-cell help, serve an important function to limit inflammation and to prevent development of autoimmune disease via clearance of self-antigens (15–17). We analyzed production of antibodies and cytokines by MZ-like B-cells, and found that among circulating B-cells they are the main producers of IgM after toll-like receptor ligation with CpG-B ODN, and that these cells also are potent producers of TNF and IL-10. While MZ-like B-cells from patients with autoimmune vasculitis and HC did not differ in functions, we found significantly reduced proportions of both MZ-like and B1-like B-cells during active disease compared with HC. Further, in the group of active treatment-naïve patients we observed negative associations between MZ-like B-cells and the levels of IgG-ANCA in the circulation, and B1-like B-cells and BVAS. However, due to the limited number of treatment-naïve patients, we need to be cautios when interpreting these results. Nevertheless, a study in SLE showed CD27^+^IgD^+^ B-cells (of which the majority expressed IgM) to be reduced in the circulation of patients, and to be inversely related with the levels of autoantibodies (28). Other studies in SLE also propose IgM to be protective, and shows that a lower ratio of IgG to IgM anti-dsDNA antibodies correlate negatively with glomerulonephritis [7]. In line with these observations, a study using a modulated mouse model of SLE, where B-cells could no longer secrete IgM, resulted in elevated levels of autoantibodies against double-stranded DNA and histones leading to a more severe autoimmune disease (29). Considering other autoimmue diseases, there is an increased frequency of several autoimmune diseases in patients with selective IgM deficiency (30), supporting a preventive role in general for IgM.

The presence of autoantigen can influence the development of autoantibodies. Neutrophil extracellular traps (NETs) display both PR3 and MPO, and has been shown to link innate and adaptive immunity through the generation of pathogenic ANCA (31, 32). It is therefore considered important that the presence of NETs is tightly regulated (33). PR3-specific B-cells and anti-MPO/PR3 autoantibodies are, however, present also in healthy blood donors (24, 25), suggesting this to be protective. Further, we have observed PR3-ANCA to negatively correlate with PR3 (34) and NETs (35) in the circulation of patients in remission, supporting a role for autoantibodies in antigen clearance. Interestingly, the proportion of PR3-specific B-cells in patients, but not in HC, increases during B-cell maturation to SwMe B-cells and plasmablasts (24), indicating an altered autoantibody repertoaire in the patients and break of immunological tolerance to PR3. Indeed, ANCA in patients predominantly consists of IgG1 and IgG4 (36, 37), meaning that B-cells have received T-cell help and undergone class-switch and affinity maturation (6). Thus, the varying features of autoantibodies instead of the presence of autoantbodies *per se* is important to consider. Concerning this, our study propose that an altered repertoaire of innate-like B-cells, which produce autoantibodies of primarily IgM class, could predispose for development of pathogenic autoantibodies of IgG class instead.

Dysfunction in other regulatory compartments in addition to innate-like B-cells, such as regulatory B (Breg) and regulatory T (Treg) cells, as well as a genetic predisposition, appear to be important for disrupting immunological tolerance and development of autoimmune disease (38–41). In this study, we did not observe a difference in IL-10 production, nor the TNF/IL-10 ratio, by the various B-cell subsets when comparing patients and HC. We cannot, however, exclude that there are other dysfunctional regulatory mechanisms concerning, for example, contact dependent mechanisms, or production of other immune regulatory cytokines. Also, as our primary interest for the functional studies was to study MZ-like B-cells, which express high amount of TLRs, we used TLR stimulation to induce cytokine production, and do therefore not know how the cells would respond to stimuli that would drive differentiation of Breg cells (42).

As expected, previous RTX treatment had a pronounced and long-standing effect on the frequency of B-cell subsets. When excluding RTX treated patients, patients in remission did no longer show reduced proportions of MZ-like B-cells compared with healthy controls. Prednisolone treatment in our study mainly affected the B-cell numbers whereas the distributions of B-cell subsets were in general unaffected. An effect of immunosuppressive treatment on the numbers of MZ-like B-cells, and other B-cell subsets, has also been described before (43). While treatment can explain differences between patients in remission and HC in our study, this is not the case for patients with active disease. The group of active treatment-naïve patients showed very similar results as the whole group of active patients, making us comfortable that we can rely on the data with respect to influence of treatment.

In conclusion, MZ-like B-cells are main producers of IgM after CpG-B ODN stimulation. We found reduced frequencies of MZ-like and B1-like B-cells in the circulation of patients with autoimmune vasculitis. This suggests a protective role of innate-like B-cells in autoimmune disease.

## Author contributions

DA, PE, JE, and MS designed experiments; DA performed experiments; DA, MS, PE, and JE analyzed data. DA and MS wrote the paper in assistance with PE and JE.

### Conflict of interest statement

The authors declare that the research was conducted in the absence of any commercial or financial relationships that could be construed as a potential conflict of interest.
