# Supplementary material for: Marginal-Zone B-Cells Are Main Producers of IgM in Humans, and Are Reduced in Patients With Autoimmune Vasculitis
Source: Front Immunol. 2018 Oct 2;9:2242. doi: 10.3389/fimmu.2018.02242 (PMC6190848; doi:10.3389/fimmu.2018.02242)
Supplement: Supplementary file 1 [file Data_Sheet_1.docx]

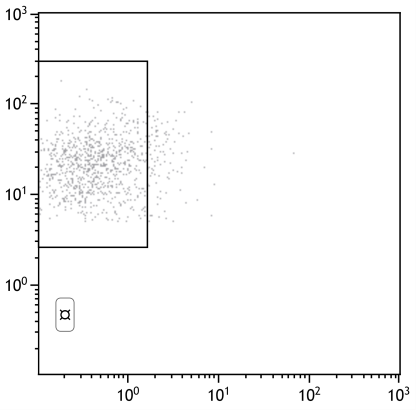

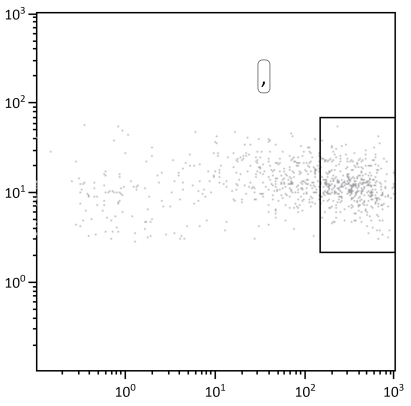

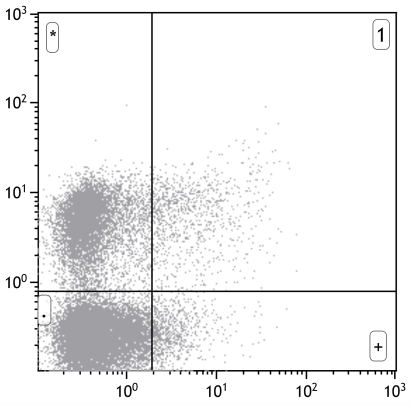

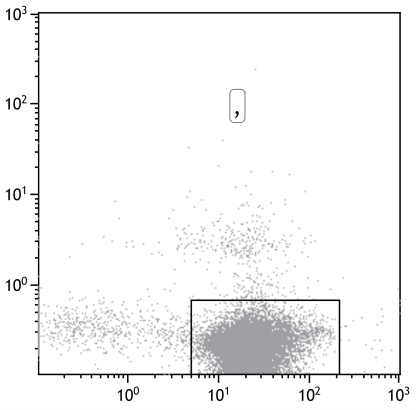

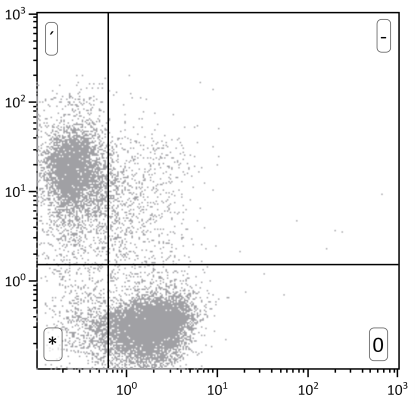


IgD

CD19

IgM

NSM

B-cells

CD27

NSM

SwMe

NSM

DN

Naive

34%

55%

6.3%

5.7%

3.0% of B-cells

CD27^+^IgD^+^IgM^high^

50%

CD3

B-cells

CD3^-^CD20^+^ B-cells

CD3^-^CD20^+^CD27^+^CD43^+^ B-cells

CD3^-^CD20^+^CD27^+^CD43^+^CD70^-^

(B1-like B-cells)

CD70

CD19

CD43

CD20

3.2% of B-cells

90%

3.7%

3.3%

63%

29%

CD27

**A**

**B**

(MZ-like B-cells)

**Fig. S1. Flow cytometry gating strategies for innate-like B-cells.** The heading on each dot-plot is the parent-population from which the cells within that dot-plot are derived from. **(A)** Marginal zone (MZ)-like B-cells were gated as CD27^+^IgD^+^IgM^high^. **(B)** B1-like B-cells were gated as CD3^-^CD20^+^CD27^+^CD43^+^CD70^-^. SwMe; switched memory; DN, double negative; NSM, non-switched memory.

**

**

**Fig. S2. Distribution of B-cell subsets in the blood circulation**

**(A-D)** Patients with active disease exhibited a reduced percentage of switched memory B-cells within the B-cell population compared with healthy controls (HC). **(E-H)** Absolute numbers of both switched memory B-cells and non-switched memory B-cells were reduced during remission and active disease compared with HC. Kruskal-Wallis test followed by Dunn’s multiple comparison test was used to compare more than two groups with independent observations (A-H). Bars indicate median and interquartile range. *p<0.05, **p<0.01, ***p<0.001, ****p<0.001

**

**

**Fig. S3. Spontaneous production of TNF and IL-10 by B-cells subsets**

Spontaneous production of TNF and IL-10 by B-cells from healthy controls (HC) was primarily observed by marginal zone (MZ)-like and switched-memory (SwMe) B-cells. Mann-Whitney U test was used to compare two groups with independent observations Bars indicate median and interquartile range. *p<0.05, **p<0.01

## Table S1. Comparison of B-cell subsets in active treatment-naïve AAV patients with HC

|  | **% of B-cells**  ***median* (IQR)** | | | **Absolute numbers**  ***x 10^6^/l (IQR)*** | | |
| --- | --- | --- | --- | --- | --- | --- |
| **Subsets** | **Active**  **(*n*=12-14)** | **HC**  **(*n*=30-31)** | **p-value** | **Active**  **(*n*=10-12)** | **HC**  **(*n*=30-31)** | **p*-*value** |
| SwMe | 18.7  (12.3-29.0) | 32.6  (20.3-41.7) | **0.0056** | 15.5  (9.7-31.5) | 31.8  (27.6-42.3) | **0.0052** |
| Naive | 55.5  (40.0-69.6) | 50.1  (37.9-55.9) | 0.1835 | 61.5  (20.1-74.1) | 40.4  (32.7-87.8) | 0.9236 |
| NSM | 4.9  (1.5-6.1) | 6.7  (4.4-10.4) | **0.0290** | 3.9  (1.0-7.1) | 7.1  (4.8-13.0) | **0.0173** |
| DN | 15.9  (12.4-25.2) | 10.9  (8.5-15.0) | **0.0041** | 13.4  (6.0-33.6) | 13.1  (9.5-15.7) | 0.6971 |

AAV, ANCA-associated vasculitis; HC, healthy controls; SwMe, switched memory; NSM, non-switched memory; DN, double negative; IQR, interquartile range. Mann-Whitney U test was used to compare two groups with independent observations. p<0.05 was statistically significant.
